# Addressing the criticisms and critiques of positive psychology: recommendations for improving the science and practice of the field

**DOI:** 10.3389/fpsyg.2025.1548612

**Published:** 2025-03-26

**Authors:** Jaclyn Gaffaney, Stewart Ian Donaldson

**Affiliations:** Division of Behavioral and Organizational Sciences, Claremont Graduate University, Claremont, CA, United States

**Keywords:** positive psychology, wellbeing, critiques, theory, checklist, practice, evaluation

## Abstract

This article presents two checklists designed to help researchers, practitioners, and evaluators address common critiques of Positive Psychology (PP) identified in a recent systematic review. These critiques focus on PP's theorizing, methodology, and its perception as a decontextualized, capitalistic endeavor. The checklists offer practical recommendations for improving future research and practice, with one tailored for researchers and the other for practitioners and evaluators. Key focus areas include self-reflection, cultural sensitivity, methodological diversity, collaboration, and ethical considerations. By acknowledging past critiques and offering concrete solutions, this paper aims to foster a more inclusive and rigorous future for PP.

## Introduction

Since its formal introduction in 1998, Positive Psychology (PP) has sought to balance traditional psychology's focus on pathology by studying human strengths, wellbeing, and optimal functioning (Seligman and Csikszentmihalyi, 2000). Over the past two decades, PP has contributed to a growing body of research on topics such as happiness, resilience, meaning, and character strengths, influencing both academic inquiry and applied interventions across various domains, including education, healthcare, and organizations.

Despite its contributions, PP has been the subject of significant critique. Critics argue that the field lacks proper theorizing and conceptual clarity, suffers from methodological shortcomings, and at times overstates its claims (van Zyl et al., 2023). Some have asserted that PP is a pseudoscience, lacks novelty, or has distanced itself from mainstream psychology. Others have suggested that PP embodies a decontextualized, neoliberal ideology that promotes individualism while neglecting social, cultural, and systemic influences on wellbeing. Additionally, some view PP as a capitalistic venture that commodifies happiness and wellbeing (Lomas et al., 2021; van Zyl et al., 2023).

As co-authors of the van Zyl et al. (2023) systematic review of these critiques and criticisms, we are invested in further exploring their implications. That review synthesized critiques related to PP's theorizing, methodology, and broader societal framing, which are summarized in Table A1 in Appendix A. This table provides a detailed overview of the critiques, their subcomponents, and how they inform the focus of this paper.

### History and context of the criticisms and of positive psychology

Founders and leaders in the field of PP have responded to critiques in various ways, with some questioning the veracity of the claims, some providing alternative perspectives and data, and some emphasizing the importance of staying focused on the peer-reviewed science in top-tier PP journals and the improvements made over time (e.g., see Seligman, 2018a,b). For example, Seligman (2018b, p. 266) acknowledges criticism in science is a main driver of progress, and admits he has “always welcomed critics and shunned ass-kissers.” However, he also argued that many criticisms of PP have been weak or even baseless, coming from critics who have not done their homework. He provided a list of common weak criticisms of PP, pointing out the faulty logic and sloppy data used to assert them. Donaldson (2022) stressed the importance of examining the criticism and critiques of the science of PP carefully, but also showed that many of these proposed limitations were not focused on, or informed by, the peer-reviewed positive psychological science literature published in the top journals. Instead, when you look under the hood, stories published in the popular press, blogs, and non-peer reviewed books and books chapters were often the aim of the attacks. Csikszentmihalyi (2020) provided a historical and anthropological perspective on the development of PP and a positive worldview, which suggests many of the critiques and criticisms are not well informed by the overall purposes for a science of psychology, and why it is much more than pseudoscience, decontextualized neoliberal ideology, and a capitalistic venture.

Further, many of the critiques and criticisms may merely be dated. Given that the field is only 25 years old, it has come a long way but still has a ways to go. As Lomas et al. (2021), Lomas and Ivtzan (2016a) have explained with their metaphor of waves of PP, the first decade was merely a reorienting of the field to study positive topics, and studies may have oversimplified wellbeing. The next decade began integrating PP with mainstream psychology and removing the false dichotomy of the positive vs. negative divide (Lomas and Ivtzan, 2016a). We are now in the third wave, adding greater complexity by considering the whole ecological system, cultural differences, and diversifying methods, thereby addressing many of the current criticisms (Lomas et al., 2021).

### Purpose of this paper

Despite progress in the field, critiques of PP continue to shape its perception and influence its credibility in research and practice. While some critiques stem from outdated views, others highlight areas that warrant further attention. Regardless of their accuracy, these criticisms remain part of the discourse around PP and affect how the field is received.

In response to these critiques, this paper takes a forward-thinking approach by introducing two checklists—one developed for researchers, and another developed for practitioners and evaluators—to help navigate and proactively address these concerns. These checklists acknowledge past critiques, preempt future criticisms, and provide practical recommendations for strengthening the science and application of PP. By doing so, we aim to foster a more rigorous, contextually aware, and ethically grounded future for the field while shaping a constructive and forward-looking narrative.

### Checklists for designing future positive psychology studies and interventions

Checklists are valued in research and evaluations for enhancing validity, reliability, and credibility (Scriven, 2005, 2007; Stufflebeam, 2000). They also provide a structured approach for PP professionals, including anyone researching, designing, or evaluating PPIs. By using the checklists at various stages and for different purposes, PP professionals can enhance the rigor, effectiveness, and impact of their work. Ultimately, the checklists help people think about the possible critiques as they develop new work and offers possible solutions based on the best science and practice knowledge we have to date.

The authors employed the first several stages of Stufflebeam's (2000) Checklists Development Checklist (CDC) to develop both checklists. The CDC is a tool that helps others create more valid checklists. The two checklists are as follows:

- **Checklist #1**: Strategies for Researchers to Design Future Positive Psychology Studies that Mitigate Criticisms (Figure A1 in Appendix C).
- **Checklist #2**: Strategies for Practitioners and Evaluators to Develop and Evaluate Future Positive Psychology Interventions that Mitigate Criticisms (Figure A2 in Appendix D).

A description of how to use the checklists can be found in Appendix B, and the full Checklists can be found in Figure A1 in Appendix C, and Figure A2 in Appendix D. Also reference the final column in Table A1 in Appendix A to see how each checklist component maps back on to the original critiques and criticisms of PP.

## Literature review - theoretical foundations for the checklist

The following outlines the theoretical foundations for each checklist and its checkpoints. Each of the two checklists is comprised of sections that categorize the content, (e.g., Self-Reflection & Development) and within those sections are the checkpoints that explicate the recommendations (e.g., Self-Awareness & Critical Self-Evaluation). The following content explains the theory and research behind each checkpoint in relation to both researchers (R) and practitioners (P). The headings fall in line with what can be seen in the checklists so checklist users can reference the literature in this section while using the checklists. The alignment between the critiques and the checkpoint recommendations can be found in Table A1 in Appendix A.

### Self-reflection and development

#### Self-awareness and critical self-evaluation (R & P)

Engaging in self-awareness and critical self-evaluation, including reflexivity, self-reflection, and critical self-evaluation, enhances awareness and addresses potential criticisms, such as poor theorizing (Theme 1) and decontextualization, which can harm marginalized groups (Theme 5). Self-examination and reflection have been practiced throughout history, from ancient philosophies to modern journaling and therapy (Foucault, 1988). Reflexivity involves examining one's research paradigm, positionality, personal biases, values, practices, and foundational beliefs to understand their influence on research questions, methodologies, and interpretations (Jacobson and Mustafa, 2019; Peters et al., 2022; Wissing, 2022).

Research paradigms, systems of beliefs guiding methodology (Morgan, 2007), shape approaches to research problems and involve reflection on ontological, epistemological, and axiological assumptions (Shannon-Baker, 2016). For example, a constructivist perspective acknowledges multiple realities and the role of biases in interpretation (Wong, 2011). As another example, for a researcher with a transformative orientation, social justice is not just a design approach but the core purpose of the research. Reflexivity also involves challenging assumptions and making positionality explicit, such as through exercises like social identity mapping, which can provide context for readers and practitioners (Jacobson and Mustafa, 2019).

Both researchers and practitioners in PP should recognize the value-laden nature of the field and transparently address how their personal values and those intrinsic to the field may steer their work. This awareness of one's positionality is crucial for self-exploration and can be reported in research to provide context for readers (Jacobson and Mustafa, 2019). These practice enhances validity, ethical grounding, and sensitivity to diverse cultural contexts.

#### Cultural competence and power dynamics (R & P)

Enhancing cultural competence and understanding power dynamics are essential to ensure respect and sensitivity toward diverse cultural contexts (Christopher and Howe, 2014; Pedrotti, 2014, 2015). These practices are critical to mitigating the criticism that PP decontextualizes and can cause harm to marginalized groups (Theme 5). The critical theory work of Foucault (1977) demonstrated how power and knowledge are intertwined. Certain institutions (like prisons and schools), produce and control knowledge to reinforce power structures. At the intersection of self-reflection and power dynamics, Foucault also posits how self-reflection of one's own power structure can help lead toward change.

More globally, post-colonialism, considering the societal implications of colonialism, and decentering Western perspectives can help inform power dynamics in research. As added nuance, Bhabha's (1994) concept of “hybridity” argues that there is no binary between colonizer/colonized since cultures interact and impact one another. By considering these power structures and cultural interplay, it is also important for researchers and practitioners to be aware of how PP might be misused as a tool for control or to endorse specific social norms, whether knowingly or not. Furthermore, it is imperative for researchers to conscientiously navigate the power structures and societal dynamics that may influence both the direction and the reception of their work.

In addition to understanding their own values and beliefs, as noted above, researchers can recognize and understand the values and foundational beliefs of other cultures, particularly those that may be impacted or involved in their study areas. For practitioners' intervention design and implementation this involves not only being aware of the cultural contexts in which they operate but also actively seeking to understand and integrate this awareness into their practice. Additionally, practitioners and those evaluating interventions should consider engaging with standards and tools that promote cultural competence. Examples include the American Evaluation Association's (2011) standards for cultural competence and the Cultural Competence of Program Evaluators self-report scale (Dunaway et al., 2012).

By adopting these approaches, researchers and practitioners can ensure that their work is culturally competent and ethically sound, thereby enhancing the overall impact and relevance of their contributions to the field.

#### Intellectual humility (R & P)

Another key component is intellectual humility, a recognition of one's own intellectual limitations (Porter et al., 2022). Embracing intellectual humility can address several critiques facing PP, including inadequate theorizing (Theme 1), unsupported claims (Theme 3), a lack of integration with mainstream psychology (Theme 4), and insensitivity to context and culture (Theme 5). A forebearer of intellectual humility is Gadamer's (1989) “fusion of horizons.” Horizons are representative of one's own vantage point, and fusing horizons involves integrating one's own perspective with the historical and cultural context of others, which may also involve engaging in dialogue with people with differing perspectives. Therefore, incorporating intellectual humility involves actively seeking out and considering viewpoints that might challenge or refute personal beliefs. Intellectual humility can also be demonstrated by valuing and engaging with diverse perspectives and maintaining openness to receiving and constructively responding to feedback from others (Porter et al., 2022).

Similarly, Rorty's (1989) “ironism” argued that our beliefs, values, and vocabularies are contingent upon our historical and cultural contexts and are not absolute truths. Researchers and practitioners can cultivate intellectual humility by acknowledging the limits and potential fallibility of their knowledge and expertise. This mindset involves remaining open to new evidence and perspectives that may either support or challenge their existing beliefs, theories, or findings. This approach not only strengthens the robustness of one's work but also fosters a culture of continuous learning and adaptation.

These qualities of fallibility, respectful engagement, and constructive dialogue are particularly crucial when working with marginalized groups to help mitigate the criticism that PP causes harm to marginalized groups by not taking their perspectives into account (Theme 5). By adopting intellectual humility, researchers and practitioners in PP can enhance the ethical integrity and effectiveness of their work, ensuring it is both scientifically robust and culturally sensitive.

#### Methodological expertise and professional development (R)/scientific literacy and professional development (P)

Professional development, scientific literacy, and active engagement are crucial for both researchers and practitioners to address criticisms about the field's scientific rigor (Theme 2), replicability issues (Theme 3), and context neglect (Theme 5). Staying informed about the latest developments in research and practice and maintaining a critical perspective toward emerging trends are key. “Enhancing the quality of professional knowledge and its application” is highlighted in Jarden et al. (2021, p. 11) ethical guidelines for PP. Both researchers and practitioners should be adept at differentiating between sound scientific methods and pseudoscience (Curd and Psillos, 2013; Daempfle, 2013). This requires regular inquiry and the re-evaluation of one's research or practice. For researchers, this professional development may involve investing in methodological training to be able to effectively employ appropriate methods. For psychologists entering the field, training in PP could enhance their work (Guse, 2010).

Lomas and Ivtzan (2016b) have called for developing guidelines for training and regulation within PP, with ideas for professional guidelines, and whether master's qualifications could lead to being called “positive psychology practitioners,” and doctorate studies leading to the title of “positive psychologists.” Vella-Brodrick (2014) similarly called for more ethical guidelines for professionalism and suggests four guiding principles for PP to consider, including integrity, industriousness, innovation, and impact. Guidelines on professionalism or accreditation standards have not been created, but ethical guidelines have been proposed (Jarden et al., 2021; more on this below).

Active participation in scholarly communities, such as the International Positive Psychology Association and its regional counterparts in Europe, Canada, Africa, India, and beyond, can be beneficial for both researchers and practitioners. Additionally, forming communities of practice or professional learning communities (DuFour, 2004) among peers can create a way to stay informed, connected, and advance professionally. These communities act as platforms for sharing insights, challenges, and advancements, enriching the collective expertise in the field. Such engagement not only fosters knowledge exchange and intellectual growth but also enhances the credibility and effectiveness of PP, ensuring it remains a scientifically robust and a contextually sensitive discipline.

### Conceptual foundations

#### Historical roots (R & P)

Understanding the historical roots of PP is essential for researchers and practitioners to situate their work within the broader context of psychological science. It is important for them to demonstrate how their work is built upon foundational scholarship from fields like humanistic psychology and ancient philosophy. This approach addresses the criticisms that PP lacks proper theorizing (Theme 1) and fails to acknowledge the contributions of past theorists (Theme 4).

Recognizing the historical and theoretical roots from which PP has developed includes acknowledging the contributions of those that predated PP. Seligman (2011) himself has recognized the foundational work of scholars such as William James, Abraham Maslow, Albert Bandura, Carl Rogers, and Victor Frankl. McMahon (2013) offers one example of the pursuit of happiness in history, and Ivanhoe (2013) offers a history of happiness in early Chinese thought. For example, Aristotle introduced the concept of “eudaimonia,” which denotes a state of living and acting well, beyond merely experiencing fleeting moments of happiness (Melchert, 2002). Similarly, the Dalai Lama interprets the Buddhist term “sukha” as a persistent state of mental equilibrium and profound understanding of life's realities (Helliwell et al., 2011).

Further, understanding the patterns of history can help educate PP professionals. For example, Kuhn's (1962) theory of paradigm shifts shows how scientific revolutions happen when new anomalies begin to create a paradigm shift from an old way of knowing to a new one. This insight can help PP professionals by offering insights into the development, acceptance, and evolution of psychological theories and practice, and understanding the dominant paradigm, navigate anomalies and innovations, integrate diverse perspectives, and adapt to new paradigms.

By placing their current research within this broader historical framework, researchers can illustrate how new ideas are not isolated but evolve from established knowledge. This contextualization is crucial as the absence of theory progression is a criterion of what constitutes pseudoscience (Curd and Psillos, 2013). Through such an informed approach, researchers and practitioners can enhance the theoretical robustness and historical awareness of their work.

#### Theoretical pluralism (R & P)/evidence-based interventions (P)

PP researchers and practitioners can seek to understand and integrate theoretical frameworks from within and outside of PP. This inclusivity can help address critiques about PP lacking proper theorizing (Theme 1), being categorized as pseudoscience (Theme 3), and its isolation from mainstream psychology (Theme 4).

Aristotle (1994) introduced the term of “multivocity,” an idea that argues that key terms within philosophy—which could extend today to the social sciences—can have multiple, context-dependent meanings. Similarly, Wittgenstein's (1953) concept of family resemblances between concepts notes that there is no single essence but rather similarities that can coexist and contribute to a richer understanding of constructs. As noted in the section above, the term wellbeing itself has contained multitudes of definitions across cultures and across time, and each context may define it a bit differently. As one example, a sample of New Zealand workers' explanations of the definition of wellbeing overlapped but converged from the common academic definitions (Hone et al., 2015).

Researchers should clearly identify and articulate the theoretical frameworks and empirical evidence informing their research. This might include social sciences theories, such as those related to leadership or behavior change, or emancipatory theories like feminist, critical race, queer, postcolonial, and indigenous theories (Creswell and Plano Clark, 2018).

Similarly, practitioners designing interventions should base their approaches on strong theoretical frameworks, empirical evidence, and insights from practice, clearly outlining the mechanisms of change (e.g., via a theory of change or logic model). Adapting interventions from other fields or incorporating various modalities into psychological wellbeing approaches can address the critique of PP being self-isolated (Theme 4). Practitioners can also utilize resources such as the unified framework for positive psychology interventions (Ciarrochi et al., 2022) and best practices from exemplary positive psychology interventions (Donaldson et al., 2021) to build evidence-based interventions.

Furthermore, instead of adhering to a single theoretical perspective, the idea of theoretical pluralism (Midgley, 2011) acknowledges that different theories can coexist, each offering valuable insights or explaining specific aspects of a phenomenon. This can mean accepting multiple valid perspectives or theories for a particular phenomenon, integrating ideas from different disciplines for a more comprehensive understanding, recognizing that different theories may complement each other even if they arise from distinct premises or levels of analysis, and being open to adapting and refining theories with new data. Therefore, researchers should consider integrating perspectives from beyond the scope of PP and evolving their theories with new evidence or new perspectives. For example, Steger updated his conceptualization of meaning in collaboration with new insights gained in a collaboration with Martela and Steger (2016).

Finally, researchers should consider incorporating theoretical frameworks from non-Western traditions, such as Buddhist psychology, Ubuntu philosophy, holistic and integrative approaches, mindfulness practices, feminist and social justice theories, and indigenous ways of knowing.

#### Construct and conceptualization rigor (R)/construct choice and terminology (P)

Researchers should engage in comprehensive literature reviews and actively seek diverse perspectives when selecting constructs and measures for their studies. This approach is instrumental in addressing critiques regarding PP's lack of integration with mainstream psychology (Theme 4). It is crucial for researchers to steer clear of the jangle fallacy, which involves using different terms for similar constructs, and the jingle fallacy, which mistakenly groups different constructs under a single label (Gonzalez et al., 2021). Avoiding these fallacies is key in countering the criticism that PP is prone to jingle-jangle fallacies (Theme 1), as exemplified in studies comparing grit and conscientiousness (Disabato et al., 2019) and PERMA with subjective wellbeing (Goodman et al., 2018).

Quine (1951) offers historical theories for PP professional's construct creation and use. Quine critiqued the analytic-synthetic distinction by arguing that there is no clear boundary between analytic statements, which are true by definition, and synthetic statements, which are true by how their meaning relates to the world. This notion could be important to keep in mind as constructs are developed in relation to other relevant constructs and that definitions should be flexible and evolve with new empirical findings. Similarly, Putnam (1975) theorized that language is interconnected with reality and that meaning of words are impacted by the external world. This means that PP conceptualizations and constructs need to be contextually grounded. Further, Hacking (1995) introduced the concept of “looping effects,” which explains that creating scientific classifications (e.g., anxiety) can influence how people understand themselves and can impact how they behave, which in turn can lead to changes in the classifications. For example, the scientific classification of anxiety can impact people's self-conceptions and behaviors and can ultimately change the classification of anxiety. Therefore, conceptualization (and subsequently, replicability) within PP will always be challenging given that constructs are ever evolving over time and across places.

Researchers can still adhere to established guidelines for creating precise conceptual definitions for their intended contexts (e.g., Podsakoff et al., 2016). Researchers should remain cautious of not forming new constructs merely by amalgamating existing ones, as advised by Newman et al. (2016). Other vital steps to ascertain that conceptualizations are appropriate and relevant involve ensuring discriminant validity from conceptually related constructs (Shaffer et al., 2016) and conducting best practices in content validation (Colquitt et al., 2019). By adhering to these rigorous standards, researchers can enhance the theoretical clarity and methodological rigor of their work. Likewise, practitioners should be meticulous in their construct choice and terminology, ensuring alignment with comprehensive literature and diverse perspectives, to avoid the pitfalls of the jangle and jingle fallacies and to maintain the integrity and relevance of their interventions.

#### Complexity in the positive (R & P)

Researchers and practitioners should recognize the multifaceted nature of wellbeing, acknowledging its complexity and the interplay of individual, societal, and systemic factors. This understanding helps address the critique that PP oversimplifies by creating a binary of positive vs. negative (Theme 1), its perceived isolation from mainstream psychology (Theme 4), and the overemphasis on individual factors (Theme 5).

While considering complexity, it is also essential for researchers to clearly define what the “positive” in PP entails, justify its application, and explain their contribution to the field. This might include engaging with Pawelski's work defining the “positive” in PP (Pawelski, 2016a,b). Practitioners can also explore this definition, along with other best practices of PP interventions (Donaldson et al., 2021; Pawelski, 2020).

Researchers and practitioners cannot create a false dichotomy of positive vs. negative. In their assertion of PP's evolution into a second wave, Lomas and Ivtzan (2016a) highlighted the need to move beyond simple dichotomies between positive and negative and incorporate complexity. They pointed out that the positive is not always beneficial; for instance, excessive happiness can be counterproductive, happiness is not universally appropriate, and its pursuit can paradoxically decrease happiness (Gruber et al., 2011; Humphrey et al., 2021). Moreover, some positive states may lead to negative outcomes, like gratitude inducing guilt, or negative approaches like anger serving as a useful motivator.

Lomas and Ivtzan (2016a) also introduced the concepts of co-valence and complementarity in emotional states. They described phenomena like posttraumatic growth and love as “co-valenced,” involving intricate interplays of positive and negative aspects. Furthermore, they observed that many aspects of life, such as love and grief, can exist in a complementary balance. This perspective aligns with Keyes' (2007) Dual Continua Model, where mental health and mental illness are separate dimensions that can intersect, allowing for the coexistence of flourishing in both mentally healthy and mentally ill individuals.

A comprehensive exploration of the positive in PP includes engaging with perspectives both within and beyond the field, embracing the complexity and nuanced interrelations of emotional states and wellbeing.

### Multidisciplinary perspectives

#### Interdisciplinary collaboration (R & P)

It is essential for researchers and practitioners to actively engage in dialogue and collaborate with experts from various fields including medicine, sociology, anthropology, political science, religion, and other social science (e.g., Emmons and Paloutzian, 2003; Wissing, 2022). By adopting this integrative and interdisciplinary strategy, PP researchers and practitioners can expand their comprehension of wellbeing. This not only enhances the field's specific research topics but also positions PP as an integrative discipline, moving away from being perceived as siloed (Theme 4).

Interdisciplinary engagement with various psychological and scientific disciplines will enrich PP researchers' perspectives and methodologies. This is particularly pertinent in addressing criticisms regarding the lack of evidence in PP and its potential contradictions with established findings in fields such as medicine (Theme 3). A collaborative approach promotes a more coherent and robust research framework, countering critiques of PP's overreliance on empiricism and positivist approaches (Theme 2) and its tendency to overlook contextual factors (Theme 5). Such an approach is instrumental in cultivating a more comprehensive and inclusive perspective within the broader realms of wellbeing and psychological research.

#### Methodological experts (R)

It is crucial for researchers and those evaluating interventions to engage with methodological specialists to enhance and validate their approach and analysis. This step could involve utilizing peer review processes to ensure reliable, valid, and credible results (Busse and August, 2021). This collaboration could involve leveraging professional networks, reaching out to authors of pertinent methodological papers, and utilizing online platforms like ResearchGate, Reddit, and Substack for expert advice. Such practices are vital in addressing concerns about PP's measurement and methodological flaws (Theme 2), as well as its challenges in replication and evidential support (Theme 3).

#### Skeptics and critics (R & P)

Researchers and practitioners can proactively engage with skeptics and critics. This engagement is essential for preventing harm (Theme 5), avoiding overstating the significance of findings (Theme 4), and addressing concerns of insufficient evidence due to tautological reasoning and the production of self-evident findings (Theme 3).

Noelle-Neumann's (1974) “spiral of silence” theory in communication posited that individuals may not always be willing to express their opinion, depending on factors related to a fear of isolation, being in a minority viewpoint, and public perception of the majority viewpoint. The spiral refers to a self-perpetuating cycle where the minority opinion continues to be silenced while the majority becomes increasingly dominant. Considering this theory, researchers, practitioners, and evaluators can solicit various viewpoints to ensure non-dominant voices are heard and integrated.

Early involvement with critical perspectives is recommended to not only refine research design but also to provide context for findings, thereby strengthening the design and bolstering the credibility of the results. Researchers should anticipate skepticism that may dismiss their questions and results as trivial, self-evident, or wrong, and prepare cogent counterarguments to underscore their work's value; this could include presenting these in research papers or reports (Booth et al., 2008). Engaging with critics not only uncovers new insights but also promotes the examination of disconfirming evidence, thereby mitigating confirmation bias—a tendency to favor findings that align with existing assumptions. Addressing this bias and engaging with external security are both crucial steps to avoid the pitfalls of pseudoscience (Curd and Psillos, 2013).

This approach is exemplified in the discourse surrounding Fredrickson's positivity ratio, where the criticism by Brown et al. (2013) and Fredrickson's (2013) response demonstrate a constructive engagement with critique. Therefore, a commitment to open, critical debate is not only a defense against such accusations but also a pathway to more robust and credible research outcomes in pp.

#### Participant involvement (R & P)

Researchers and practitioners are encouraged to collaborate, consult, or seek feedback from their target sample or participants. This recommendation addresses critiques of PP for its cultural and gender biases and its inadequate representation of marginalized groups' experiences (Theme 5). Such participant engagement ensures that research is attuned to the needs and contexts of the populations it seeks to understand or benefit. Methods like piloting studies or conducting “think alouds,” where participants express their thoughts, feelings, and opinions, can help verify that the aspects being tested are comprehended as intended (Dillman, 2007). Furthermore, researchers should consider participatory action research (Baum et al., 2006; Mehari et al., 2023) or participatory mixed methods (Olson and Jason, 2015), and evaluators of interventions should consider collaborative, participatory, or empowerment evaluations (Fetterman et al., 2018), to foster deeper engagement and more inclusive research practices.

#### Cultural perspectives (R & P)

It is important to integrate cross-cultural or culturally relevant perspectives within research teams and participant groups. PP has faced criticism for overlooking social institutions (Theme 1) and generalizing Western or WEIRD (Western, Educated, Industrialized, Rich, and Democratic; Hendriks et al., 2019) norms as universally applicable. This consideration becomes especially pertinent when conducting research with non-WEIRD and indigenous populations, to ensure that the research is inclusive and culturally sensitive (Theme 5). Scholars such as Brouwers (2018), Chaudhary (2018), Hendriks (2018), and Lomas (2020) have emphasized the importance of cross-cultural perspectives. Jarden et al.'s (2021) guidelines on PP practice also highlights “appreciating the diversity of human experience and cultures” (p. 11) that respects and celebrates people's unique paths, context, and cultures. Additionally, this approach involves actively seeking diverse viewpoints in peer review processes, contributing to the decolonization of this work, and ensuring a broader, more inclusive understanding of cultural nuances in PP research.

#### Research-practice collaboration (R & P)

Strengthening the connection between research and practice is vital for reducing the research-practice gap and addressing the critique that PP often overlooks contextual factors and marginalized groups (Theme 5).

Researchers are encouraged to form partnerships with practitioners to co-produce research that is both relevant and actionable in real-world settings, and to assist practitioners in understanding research methodologies and statistics (Rynes-Weller, 2012). Research-practice partnerships in education can serve as a model for potential collaborations in other disciplines (Coburn et al., 2013). Ultimately, such collaborations foster contextually informed and culturally sensitive research and equip practitioners with directly applicable, evidence-based strategies.

Practitioners, in turn, are encouraged to collaborate with researchers to create interventions that are empirically testable and rooted in research. This joint effort ensures the scientific validity of these interventions and enriches academic literature with insights derived from practical experiences.

### Methodological rigor in research and evaluation

#### Diverse methodologies (R & P)

PP researchers have faced criticism for overly relying on cross-correlational or experimental methods, often overlooking the value of diverse methodologies (Theme 2). To counteract superficial findings, researchers should explore advanced methods beyond basic cross-sectional and correlational studies (Lomas et al., 2021; Wissing, 2022). This includes embracing both quantitative and qualitative approaches to fully capture the complexities of positive psychological phenomena (van Zyl et al., 2019).

Feyerabend (1993) argued many decades ago against methodological monism, stating that there is not a single universal scientific method that is most applicable to all scientific inquiries, and many have followed in suit ever since. Additionally, Habermas's (1971) theory of knowledge-constitutive interests posits that there are three interests that guide inquiry and knowledge production: technical (which would map onto the empiricism of quantitative methods), practical (which would map onto qualitative methods), and emancipatory (which would map onto social justice methods), further demonstrating the variety of methods available.

Scholtz et al. (2020) highlighted that most psychology publications predominantly use quantitative methods, with a significant reliance on self-report questionnaires. This underscores the need for greater diversity in research approaches. Lomas et al. (2021) advocated for the use of more qualitative and mixed methods in the call for greater scientific progression in PP's third wave. Researchers could consider employing multiple methodologies within the same series of studies to triangulate or build from one finding to another (Creswell and Plano Clark, 2018). This mixed-methods research can provide a richer, more nuanced understanding of positive psychological phenomena. See Creswell (2021) for user-friendly guidelines on how to conduct mixed methods studies, and Creswell and Plano Clark (2018) for greater detail.

Qualitative approaches can capture the richness and nuance that quantitative measures may miss. Longitudinal and experimental designs can be critical for establishing causality and observing changes over time. Ecological momentary assessment (e.g., Shim et al., 2021) can mitigate self-report biases by collecting real-time data and reducing recall bias. Experience sampling method offers another technique for capturing momentary experiences and behaviors in natural settings. See Tamlin et al. (2003) for guidelines and Nakamura et al. (2022) for an example. Objective measures, like behavioral observations, physiological indicators, eye trackers, and hormonal response analyses (van Zyl et al., 2023) can further address biases and oversimplifications.

Participatory action-research can also play a crucial role in collaboratively creating knowledge with marginalized communities, thereby addressing the criticism of decontextualization (Theme 5). See Cornish et al., 2023 for an overview of participatory action research. Harding's (1991) standpoint theory argues that since knowledge is socially situated, marginalized populations can have valuable perspectives that could be considered a bit more objective, or at least, nuanced, given their outsider perspective. Therefore, integrating marginalized perspectives into research can provide nuance to a research process.

Plus, both researchers and practitioners can address critiques of PP's lack of novelty (Theme 4) by adopting innovative PP-specific methods like appreciative inquiry (Cooperrider and Whitney, 2000) or bright spots analysis (Heath and Heath, 2010), further diversifying and enriching the greater field of psychology's methodological repertoire.

It's essential for researchers to justify their methodological choices, recognizing that methodological standards are continually evolving with advancements. Engaging in ongoing methodological innovation and adaptability is key to staying current.

#### Methodological rigor (R)/evaluative rigor (P)

Implementing rigorous methodologies is imperative in addressing critiques concerning the validity, replicability, and evidence base of PP (Themes 2, 3). This need for rigor has been acknowledged by several scholars in the field (e.g., Goodman et al., 2021; Lomas et al., 2021; van Zyl et al., 2019; van Zyl and Rothmann, 2022).

van Zyl et al. (2019) noted many considerations for enhancing rigor in PPI research, design, and evaluation, including conducting power analyses to achieve greater effect sizes and avoiding HARK-ing (e.g., excluding data points after analyses and dropping non-significant measures). See Simmons et al. (2011) and John et al. (2012) for more on these points. van Zyl et al. (2019) also highlighted the importance of including moderators and mediators in studies to find more nuance in terms of PPI efficacy, such as understanding who benefits most from interventions, or who may potentially be harmed. In PP, this could include seeking to find a “person-activity fit” between the individuals and the appropriate PPIs (Schueller, 2014), since some activities may be more suited for some populations. For example, some interventions backfire with certain populations, such as a Siegel and Thomson (2017) study that demonstrated that gratitude exercises may leave depressed populations feeling like they do not have much to be grateful for.

Researchers and evaluators should reference and adhere to method-specific standards, such as Cochrane's risk of bias in randomized controlled trials (Eldridge et al., 2016).

Practitioners, on the other hand, are advised to employ established measures and evaluative practices in their assessments. Collaboration with expert researchers and evaluators who maintain methodological rigor and uphold standards is essential for the reliability and validity of evaluations. Engaging with professional organizations, such as the American Evaluation Association (AEA), can further enhance this rigor. Utilizing validated measures to assess intervention effectiveness also contributes valuable evidence to discern what is effective and what is not.

However, it is crucial to recognize that rigor does not equate to perpetuating a colonial view of research. Instead, it means inclusively embracing indigenous or qualitative methods, like art or storytelling, which may sometimes be undervalued in the academic community for their scientific soundness (e.g., Avery, 2023). This inclusive approach ensures that the richness and diversity of all research methods are acknowledged and utilized effectively in PP.

#### Psychometric rigor (R)

Upholding rigorous psychometric standards is essential, particularly when developing new constructs, conceptualizations, or measures. This adherence is crucial to address critiques that PP does not employ psychometrically sound methods (Theme 2). Michell (1999) called for methodological rigor and argued that psychology has relied too heavily on quantification and measuring constructs numerically and posited that using both quantitative and qualitative methods will lead to richer measures.

Researchers should also be aware of various best practices in the field for scale development and validation and perform comprehensive validation and reliability testing (Boateng et al., 2018; Cortina et al., 2020; Djurdjevic et al., 2017; Hinkin, 1995, 1998). This includes best practices in content validation (Colquitt et al., 2019) and factor analysis (Kline, 2014). Additional existing standards include the Standardization of Behavior Research guidelines (Elson et al., 2023), International Test Commission's (2017) guidelines for translating and adapting tests to other populations; the European Federation of Psychologists's (2025) guidelines on test construction, adaption, and validation; and the International Organization for Standardization's (2011) standards for assessment methods and procedures. Adhering to these guidelines ensures that the methods and tools used in PP research are not only scientifically rigorous but also culturally sensitive and universally applicable.

As noted in the Construct & Conceptualization Rigor section, many researchers rename old constructs (the jangle fallacy) and therefore create new measures where others may already exist. Similarly, some researchers make up new measures to promote their own brands instead of finding and using an existing one (Elson et al., 2023). As evidence across psychology, Elson et al. (2023) found that 43% of measures were only used in one study. PP researchers can counteract this by exploring existing options before resorting to making something new.

In both the conceptualization process (see Construct & Conceptualization Rigor) and in establishing or adapting new measures, researchers can engage with the populations they are serving and with other subject matter experts, thereby engaging in collaborative and consensus-building processes, like the Delphi method (Pearce et al., 2012) or nominal group technique (Harvey and Holmes, 2012). Additionally, conducting cross-cultural studies can aid in understanding how positive psychological constructs operate in different cultures or with different demographic groups (Lopez et al., 2019), challenging the assumption that Western norms as universally applicable (Theme 5). Both researchers and those evaluating interventions can try to use or adapt instruments that have gone through psychometric testing when they are not developing their own.

#### Replicability and transparency (R)

In the pursuit of scientific integrity, it's important for researchers to actively promote replicability and transparency in their work. Poor replicability has been found across all of psychology (Maxwell et al., 2015) and within PP specifically (for an example related to the positivity ratio, see Friedman and Brown, 2018). Unfortunately, questionable research practices are also common (John et al., 2012).

Our notions of what is true evolves over time, which can impact replicability as well. For example, Kuhn (1962) noted how we tend to see the same data in fundamentally different ways once there is a paradigm shift. What is considered valid and replicable in one paradigm might not be seen that way in another. Additionally, scientists will never be free from their own interpretations and paradigms, which will also impact replicability. In one study where multiple data analysts examined the same dataset, the results varied widely (Silberzahn et al., 2018). These findings highlight how subjective, yet defensible analytic choices can shape research results, and demonstrates that substantial variability in the outcomes of complex data analyses is challenging to avoid, even for well-intentioned experts.

Enhancing replicability can counteract criticisms that PP is a pseudoscience (Theme 3) due to this lack of replicability (Curd and Psillos, 2013). Essential practices include facilitating external scrutiny and soliciting constructive criticism; pre-registering studies on platforms such as the Open Science Framework; publishing null or inconclusive results; openly sharing datasets, methodologies, and statistical analyses; ensuring sample sizes are sufficiently powered; and conducting replication studies, including of one's own work (Efendic and van Zyl, 2019; Shrout and Rodgers, 2018).

To avoid questionable research practices like p-hacking, phishing, and data dredging, researchers should embrace transparency and accountability. They can refer to the guidelines set by Nosek et al. (2015) for promoting an open research culture. These guidelines include minimum standards for citation plans, transparency across data, analytical methods, research materials, and research design, as well as pre-registration of studies and analysis plans, and insights for how to encourage replication studies. Additional guidelines can be found in van Zyl et al. (2019), van't Veer and Giner-Sorolla (2016), and Wagenmakers et al. (2012).

Acquiring sufficient funding and resisting the “publish or perish” pressure (van Dalen and Henkens, 2012) to publish prematurely are also important. By being transparent about research methods, data, and analyses, researchers invite external scrutiny and constructive criticism, which are vital for the continued advancement and credibility of the field.

#### Theory-driven evaluation (P)/iterative testing (P)

To enhance the efficacy and responsiveness of interventions, practitioners can align their evaluation strategies with theoretical foundations and commit to continuous refinement through iterative testing. Theory-driven evaluation is an approach to assessing the effectiveness of a program or intervention based on the theoretical principles that underlie it; evaluators examine not just whether a program achieves its goals, but also how and why it does or does not achieve them (Donaldson, 2007, 2022).

Practitioners are advised to refine their interventions through practices like piloting, ongoing monitoring, frequent assessments, and establishing feedback loops (e.g., Charvet, 1995; Meadows, 2009). This iterative process is essential for continuous improvement and optimizing intervention effectiveness. Such continuous monitoring and adaptation are crucial in addressing critiques of PP regarding inadequate theorizing (Theme 1), methodological shortcomings (Theme 2), and issues with replicability and perceptions of pseudoscience (Theme 3). Additionally, iterative testing plays a significant role in reducing potential harm (Theme 5) by identifying and addressing issues affecting marginalized groups, including women, racially diverse populations, transgender individuals, and those experiencing alienation and oppression. This approach ensures that interventions are not only theoretically sound and methodologically robust but also socially sensitive and inclusive.

### Customization to the context and ethical responsiveness

#### Context-sensitive design (R & P)

In both research and practice, it is important for PP professionals to ensure their work is contextually sensitive and culturally competent. PP has faced criticism for methodological insensitivity to cultural nuances (Theme 2), and replicability (Theme 3). Additionally, the field has been critiqued for promoting a neo-liberal perspective that overly emphasizes individual responsibility for wellbeing while overlooking cultural, environmental, societal, and structural influences such as gender, class, and ethnicity (Theme 5), and similarly, overlooking the role of positive institutions and the impact of these greater structures on an individual's wellbeing (Theme 1).

Foundational to context-sensitive design is the concept of systems thinking, which is framework that focuses on understanding the interrelationships and patterns within a system rather than viewing its components as isolated parts (Kim, 1999; Meadows, 2009). A system consists of interconnected elements that collectively produce a distinctive pattern of behavior. These interconnections function through the flow of information and feedback loops, which influence future behavior by either stabilizing, resisting, or enhancing growth. Bateson (1972) further explored the ecology of mind, a holistic perspective that emphasizes how living systems are interconnected and interdependent, noting the importance of how context, environment and relationships impact behavior. Systems-thinking helps PP professionals design context-sensitive studies and interventions by considering multiple layers and their interactions, ensuring tailored, effective, and sustainable outcomes while comprehensively understanding various influencing factors and identifying leverage points for change within the system.

Additionally, a system can encompass multiple layers, as described by Bronfenbrenner's (1986) Ecological Systems Theory of human development, which includes the microsystem (immediate environment), mesosystem (interconnections between microsystems), exosystem (external environment), macrosystem (cultural and societal influences), and chronosystem (historical events and changes over time). Sapolsky (2017) more recently took an interdisciplinary perspective to emphasize the various biological, social, and environmental components that impact behavior. Additionally, Lomas et al. (2021) suggested that PP has entered a third wave that now incorporates systems, contexts, and cultures.

To address these critiques, it's important to tailor research designs and practices to the specific contexts and samples, accounting for individual and group differences in aspects such as personality, culture, and ethnicity (Lopez et al., 2019; Pedrotti, 2014). Culturally responsive research and evaluation involves respecting and understanding participants' cultural backgrounds, actively involving them in the research process, and adapting methods to be ethical, relevant, and inclusive (Casillas and Trochim, 2015; Goghari and Kassan, 2022; Hood et al., 2015; Lemos and Garcia, 2020). This approach fosters inclusivity, collaboration with communities, and addresses power imbalances, producing outcomes that are both accurate and respectful of diverse perspectives. This could also include disaggregating data across diverse groups to explore nuance.

Additionally, researchers and practitioners can integrate perspectives and approaches from various ways of thinking and knowing. For example, building on research practices from Buddhist psychology, Ubuntu philosophy, or indigenous populations will broaden the scope and cultural sensitivity of the research and practice.

Those evaluating interventions should include a wide range of outcomes, including societal and community health, to provide a comprehensive understanding and avoid misinterpreting PP's impact and conflating individual outcomes with group outcomes (Theme 1). Additionally, the experiences of marginalized or underrepresented groups, such as expatriates and the LGBTQ community, should receive focused consideration to mitigate the criticism that PP can cause harm to such groups (Theme 5).

Practitioners can acknowledge and understand structural barriers faced by participants in their wellbeing pursuits. By validating the unique experiences of individuals within groups, practitioners can ensure that their work is sensitive to the complex interplay of various factors impacting wellbeing. This may involve advocating for policies that promote wellbeing at a societal level, rather than focusing solely on individual change.

Along these lines, researchers and practitioners can both incorporate multi-level, systems-thinking approaches (e.g., individual, group, organizational) and examine the interplay among these levels (Kern et al., 2019; Lomas et al., 2021). This could involve thinking about research or an intervention in terms of bio-psycho-social-ecological wellbeing (Wissing, 2022). This approach can also mitigate the criticism that PP ignores context (Theme 5) and abstracts individual to organizational or societal outcomes (Theme 1).

#### Ethical responsibility (R & P)

Researchers and practitioners have an ethical obligation to preemptively address potential concerns in their work. This will mitigate the critiques of decontextualization and harm to marginalized groups (Theme 5). For researchers, this includes assessing the broader, long-term societal impact of their research and findings, especially considering the potential for harm and unintended consequences. Similarly, practitioners must consider the effects of their interventions on participants, their contexts, and society at large. Professionals can consider the theory of utilitarianism, also known as the “greatest happiness principle” as developed by Mill (1863) that posited that actions are morally right if they foster the greatest happiness for the greatest number of people. More recently, Kern et al. (2019) introduced systems-informed positive psychology which posed a similar resolution that wellbeing is everyone.

Adhering to established ethical guidelines is paramount. Researchers can follow the American Psychological Association's (2016) code of ethics, which outlines principles including beneficence and non-maleficence, fidelity and responsibility, integrity, justice, and respect for people's rights and dignity. Additionally, researchers can review van Zyl et al.'s (2019) best practice guidelines for PPI research design, which calls for more effective research related to “(a) intervention design, (b) recruitment and retention of participants, (c) adoption, (d) issues with intervention fidelity and implementation, and (e) efficacy or effectivity evaluation” (p.1).

Practitioners can refer to Jarden et al.'s (2019, 2021) ethical guidelines for PP practice, which encompass principles of beneficence/non-maleficence, responsible caring, respect for people's rights and dignity, trustworthiness, justice, and autonomy. Version 2.0 (Jarden et al., 2021) explicates the values of “Protecting the safety of clients and others, Alleviating personal distress and suffering, Ensuring the integrity of practitioner-client relationships, Appreciating the diversity of human experience and culture, Fostering a sense of self that is meaningful to the person(s) concerned, Enhancing the quality of professional knowledge and its application, Enhancing the quality of relationships between people, Increasing personal effectiveness,” and “Striving for the fair and adequate provision of counseling, psychotherapy, coaching, and wellbeing services” (p. 10). For example, “protecting the safety of clients and others” explains how practitioners should avoid causing harm (Theme 5) by remaining aware of how clients respond to services and seek expertise beyond one's own abilities. These guidelines also point to several other existing guidelines for practitioners to explore (see Jarden et al., 2021 Appendix G, p. 38), such as the British Association for Counseling and Psychotherapy's ethical framework for good practice in counseling and psychotherapy.

Further, the International Positive Psychology Association (2022) developed a professional code of conduct that includes the following topics: be respectful, respect intellectual property, respect diversity, be accurate, be just, be transparent, adhere to professional standards, adhere to high scientific standards, adhere to high practitioner standards, non-endorsement, scope of power, conflict of interest, membership confidentiality, respect, and termination of membership.

Additionally, evaluators of interventions can adhere to the American Evaluation Association's standards, focusing on utility, feasibility, propriety, and accuracy (Yarbrough et al., 2011). Lastly, both researchers and practitioners should commit to promoting diversity, equity, and inclusivity in their work. This includes referencing the American Psychological Association's (2019) guidelines on race and ethnicity in psychology to ensure that their work is not only ethically sound but also culturally sensitive and inclusive.

#### Conscious funding choices (R & P)

Both researchers and practitioners in PP should be discerning about their funding sources, given the field's critiques concerning its potential use as a capitalistic tool for the medicalization and commercialization of positivity (Theme 6). Researchers should be aware of funders' commercial interests, ensuring that these do not compromise the integrity and objectivity of their work. Practitioners, particularly those in commercial settings, can critically evaluate the commercial aspects of their practice, ensuring the integrity of their investor sources and contemplating partnerships with non-profit organizations. Additionally, transparency in financial matters, such as disclosing earnings from presentations related to their work (Chivers, 2019), not just the funding sources of research, can help maintain ethical standards and public trust.

### Balanced and accessible reporting and communication

#### Balanced communication and responsible claims (R & P)

Researchers and practitioners can ensure their communication is accurate, contextually grounded, and sets realistic expectations. Booth (1961) introduced the idea of showing (presenting what happened) vs. telling (the narrative explanations) that can be brought into this context. Researchers and practitioners need to distinguish between showing and telling to avoid conflating interpretations and recommendations with presenting the facts. This also allows the audiences, and people from various perspectives, to add their own interpretations. This could include making full data collection tools available for review and being nuanced and transparent in the way that findings are reported.

Researchers should base their conclusions on robust findings, exercising intellectual humility and judicious use of causal language. They should articulate both the strengths and limitations of their research, without overstating significance, overpromising impact, or overgeneralizing to other populations outside of the sample. This might involve acknowledging the specific sample and context in which a study was conducted and reporting findings in the past tense (Peters et al., 2022), while avoiding inferring patterns beyond the data to tell a story (Williams et al., 2013). This approach counters the critique that PP exaggerates claims and overstates impact (Theme 4) and sells the pursuit of happiness and unattainable dreams (Theme 6). Additionally, researchers can embrace scrutiny and constructive criticism, publishing findings that both confirm and disconfirm original hypotheses to address concerns about replicability (Theme 3).

Practitioners can similarly avoid overstatements and transparently market their services, setting realistic expectations and emphasizing the complex process involved in fostering wellbeing. They can avoid contributing to the idea that happiness is a simple commodity.

Both groups should refrain from projecting individual results as indicative of broader systemic outcomes and resist oversimplifying personal experiences as reflective of group or societal conditions without adequate theoretical support. Both groups can instead explain the context and the various aspects of bio-psycho-social-ecological wellbeing (Wissing, 2022) when setting up a study and/or explaining an intervention or findings. Addressing these nuances can counter critiques of PP being overly prescriptive, ignoring the complexities of life (Theme 1) and its focus on internal attributions while creating social oppression by pathologizing those who do not fit within the optimal criteria of flourishing (Theme 5).

#### Accessible dissemination (R & P)

Researchers and practitioners can strive to make their research and interventions widely accessible to counteract the perception of PP as a capitalistic venture focused on profiting from the pursuit of happiness (Theme 6). Accessible participation and dissemination not only challenge the notion that happiness is a commodity available only to those who can afford it but also ensures that PP practices do not inadvertently harm marginalized groups (Theme 5). There can be a significant financial cost associated with the industry of happiness, including psychological assessments, consultancy, and self-help materials, which can be viewed as medicalizing (Thompson, 2018) and monetizing positive experiences (Theme 6).

Perelman and Olbrechts-Tyteca's (1969) New Rhetoric theory introduced the idea that there is no universal audience nor any universally valid arguments, and that effective arguments should consider the “particular audience” and their values, beliefs, expectations, and contexts. Additionally, they posited incorporating a dialectical method that includes different perspectives. Additionally, Freire (1970) introduced the concept of “conscientization” in relation to pedagogy. It involves both reflection and action: deep, critical reflection of oneself, one's social reality, and one's power and oppression, paired with taking action to create social change. Both theories underscore the need for considering many viewpoints when disseminating and messaging interventions, knowledge, and findings.

Jarden et al.'s (2021) ethical guidelines for PP practitioners urges this point with the value of “striving for the fair and adequate provisions of PP services” (p. 12). Key strategies include minimizing costs, employing clear and understandable language, and addressing structural barriers to wellbeing. Innovative dissemination methods like data walks, inclusive data visualization (e.g., Guetterman and Fetters, 2022; Stephanie Evergreen Data Visualization Checklist, 2016; Tay et al., 2018), and storytelling (Knaflic, 2015) can further enhance accessibility and engagement.

For researchers, utilizing open access journals is a practical method to reduce financial barriers for audiences (though that financial burden can then fall back on researchers that may not have funding for this step). Collaborating with media and the public is crucial for accurately representing research findings and the science behind interventions. Further, they can aid practitioners in understanding and applying these accessible dissemination techniques effectively. Practitioners can create interventions that are either affordable or free, and ensure they are accessible to diverse socioeconomic groups (Donaldson et al., 2021). They can offer free resources and tools, making wellbeing practices more accessible, regardless of their financial status.

## Discussion

This paper offers two checklists to help researchers and practitioners think through the potential criticisms and critiques of their work prior to finalizing new research or intervention designs. It is our hope that the checklists represent a significant step forward in helping positive psychologists address critical issues within PP research, evaluation, and practice. Our paper highlights the importance of methodological diversity, cultural competence, ethical responsibility, and interdisciplinary collaboration. The checklists emphasize the need for researchers and practitioners to engage in continuous self-reflection, critical evaluation, and professional development. The incorporation of historical roots, theoretical pluralism, and a focus on the complex nature of positive phenomena ensures that PP research and interventions remain grounded, inclusive, and contextually sensitive.

Other frameworks to strengthen future PP research and practice (e.g., Jarden et al., 2021; van Zyl et al., 2019) have also been offered, and their suggestions have been integrated throughout this paper. Others have responded to the criticisms as well (e.g., Alexander et al., 2024; Van Tongeren, 2024; Worthington, 2024). No single set of guidelines can fully capture the complexity of the challenges faced in PP research and practice. However, checklists specific to the critiques and criticisms of the field provides a useful lens for researchers, practitioners, and evaluators. Lomas et al.'s (2021) third wave of PP called for greater complexity, methodological diversity, and contextual relevance. The checklists help guide PP research and practice in this third wave.

The recommendations provided herein are not merely a response to the critiques but a proactive step toward shaping a more nuanced and robust narrative around PP. By acknowledging and addressing both historical and contemporary criticisms, we aim to foster a more balanced and critical discourse within the field. Our intention is that these efforts will contribute to the development of a more sophisticated and inclusive PP, one that is better equipped to navigate the complexities of human experience and the diverse needs of global communities.

### Theoretical implications

This framework acknowledges the evolutionary trajectory of PP, recognizing its growth and the need for continued adaptation. By suggesting a diverse range of methodologies and emphasizing cultural competence, the paper contributes to the maturation of PP as a discipline that is increasingly inclusive and reflective of varied human experiences. The call for interdisciplinary collaboration encourages the infusion of diverse perspectives, which could lead to novel theoretical models that encompass a broader spectrum of human experiences and wellbeing. The paper's emphasis on a critical eye of the critiques suggests a dynamic and self-corrective approach within PP. This is essential for the field's credibility, as it demonstrates a willingness to engage with and learn from criticism, a hallmark of a maturing science.

### Practical implications

Providing practical checklists for researchers and practitioners serves as a valuable tool, guiding the design and evaluation of PP studies and interventions. These checklists can help standardize practices, ensuring they are grounded in the latest research and ethical considerations. Jarden et al.'s (2021) ethical guidelines for practice noted that a future iteration or section could include ethical guidelines for conducting PP research. Though this paper is not comprehensive of all possible ethical guidelines for PP research, it includes seminal information that are important to mitigating the critiques of the field. Jarden et al.'s (2021) guidelines also discussed working toward a “Code of Ethical Positive Psychology Practice,” and we think that the contents within this paper will help inform that future code.

### Limitations and future directions

The recommendations and frameworks provided are informed by the specific critiques and criticisms identified in the van Zyl et al. (2023) systematic review. While this focus ensures a thorough examination of known issues, we also encourage an ongoing, dynamic dialogue to incorporate emerging challenges and criticisms within the field of PP into this conversation, and ultimately into the checklists. These checklists are designed as living documents, intended to be refined through expert review and validation. Future research and practice will benefit from these evolving checklists, inviting continuous improvement and adaptation to new insights.

The checklists themselves already acknowledge the various ways of knowing and the multitude of perspectives and methods that can be relevant. In this vein, we recommend that journals, researchers, evaluators, and practitioners who adopt the checklists continue to have an open mind in terms of diverse perspectives, theories, and methodologies. We also acknowledge that researchers operating outside the recommendations of these checklists can still make substantial contributions, demonstrating that adherence to these criteria is not the sole measure of impactful research or practice.

## Conclusion

This paper offers two checklists that provide a guide that can be used to prevent the criticisms and critiques of PP outlined in the systematic review by van Zyl et al. (2023). Through forward-thinking recommendations, we aim to bridge the gap between past criticisms and future advancements, contributing to the evolution and growth of PP. The development of two distinct checklists—one for researchers and another for practitioners and evaluators—underscores our commitment to enhancing the rigor, relevance, and impact of PP research, evaluation, and practice. This paper serves as a call to action for researchers, evaluators, and practitioners to engage in a reflective and dynamic process of growth, collectively elevating the science and practice of PP. As the field continues to evolve, it is essential to revisit and revise these recommendations, ensuring that PP remains a dynamic, inclusive, and impactful discipline.

## Figures and Tables

**Table A1:** Original critiques and criticisms of positive psychology and the checkpoint that represents how to address each one.

	**Critique section**	**Critique section description**	**Critique elements**	**Checklist representation**
**Theme 1: Positive psychology lacks proper theorizing and conceptual thinking**.				
	*Positive psychology lacks a unified metatheory that grounds the philosophy underpinning the science and fails to provide a clear set of ideas or criteria on how positive psychological phenomena should be conceptualized, examined, and approached*.			
1.1	Poor metatheory	PP lacks a coherent meta-theoretical perspective, including clarity on key philosophical underpinnings.	a. Lack of a set of philosophical principles b. Convoluted view of human nature c. Ideological bias (all people are inherently good) d. Lack of a theory of human development	-Self-awareness & critical self-evaluation (R, P) -Historical roots (R, P) -Theoretical pluralism (R, P) -Theory-driven evaluation (P)
1.2	Poorly conceptualized virtues	Virtues are not well-conceptualized, misaligning with traditional understandings and over-relying on empiricism.	a. Poor conceptualization of virtues b. Violation of Aristotelian philosophy (virtues are seen as neutral) c. Virtues reduced to “preferred” behaviors d. Measured as continuous e. Not grounded in theory f. Reflective elements g. No virtue ethics	-Historical roots (R, P) -Theoretical pluralism (R, P) -Construct & conceptualization rigor (R, P)
1.3	No clear definition of “positive”	The term “positive” lacks a clear and agreed-upon definition, complicating the measurement of positive qualities.	a. Conceptualization of positive b. Positivity defined by lack of positive	-Historical roots (R, P) -Theoretical pluralism (R, P) -Complexity in the positive (R, P)
1.4	The positive vs. negative divide	The field artificially divides experiences into positive or negative categories, ignoring the complexity and context-dependent nature of human emotions.	a. Artificial divide between positive and negative b. Convoluted understanding of emotions c. Adaptive vs. maladaptive d. Not useful for process of living e. Usefulness of negative emotions/experiences ignored	-Self-awareness & critical self-evaluation (R, P) -Theoretical pluralism (R, P) -Complexity in the positive (R, P)
1.5	Differences and inconsistencies in concepts/theories	There is no consensus on key constructs like wellbeing and happiness, leading to conceptual confusion.	a. Lack of agreement on conceptual definitions of constructs b. Inconsistencies in theories (e.g., VIA)	-Intellectual humility (R, P) -Historical roots (R, P) -Theoretical pluralism (R, P) -Construct & conceptualization rigor (R, P) -Interdisciplinary collaboration (R, P)
1.6	The jingle and jangle fallacy	PP conflates different concepts under the same labels and uses different terms for similar concepts, obscuring theoretical clarity.	a. Same term used for different concepts (e.g., flourishing) b. Constructs vaguely defined c. Meaning of self d. Same thing, different jackets (e.g., grit/joy)	-Intellectual humility (R, P) -Historical roots (R, P) -Theoretical pluralism (R, P) -Construct & conceptualization rigor (R, P) -Interdisciplinary collaboration (R, P)
1.7	Level of abstraction	The field abstracts individual experiences to the societal level without adequate justification, potentially misrepresenting the collective experience.	a. Individual experiences abstracted to organizational level b. Happiness to wellbeing	-Construct & conceptualization rigor (R, P) -Methodological rigor (R, P) -Balanced reporting & responsible claims (R) -Balanced communication & responsible claims (P)
1.8	Lack of theoretical grounding of interventions	Interventions lack solid theoretical foundations, resulting in inconsistent empirical study results.	a. Lack of theoretical underpinning	-Self-awareness & critical self-evaluation (R, P) -Theoretical pluralism (R, P) -Evidence-based interventions (P)
1.9	Positive institutions	There is insufficient focus on conceptualizing positive institutions, neglecting the impact of social structures on individual positivity.	a. Little focus on positive institutions b. Naive understanding of social institutions	-Theoretical pluralism (R, P) -Cultural perspectives (R, P) -Context-sensitive design (R, P)
**Theme 2: Issues with measurement and methodology**.				
	*Positive psychology shows poor operationalization and measurement of its constructs, employs flawed research methodologies, over relies on empiricism/positivism and fails to employ more robust research approaches*.			
2.1	Measurement and operationalization of constructs	There is a lack of standardization in defining and measuring positive psychological constructs, resulting in multiple instruments and inconsistent findings.	a. Multiple options to measure the same construct b. Crude measurement c. Self-report: recall bias and overinflation d. Subjective vs. objective measures e. Poor measurement of constructs f. Poorly developed psychometric instruments g. Everything correlating with everything (multicollinearity) h. Measurement of individual experiences, abstracted to group level	-Diverse methodologies (R, P) -Methodological rigor (R) -Psychometric rigor (R) -Evaluative rigor (P) -Balanced reporting & responsible claims (R) -Balanced communication & responsible claims (P)
2.2	Overemphasis on empiricism and positivistic approaches	PP prioritizes empirical, quantitative methods and is overly prescriptive, potentially oversimplifying complex phenomena and missing nuances.	a. Obsession with empiricism b. Reductionist thinking: reduction to measurable quantities c. Rush to judgment d. Oversimplified answers/reductionist thinking e. Focus on describing rather than understanding phenomena f. Prescriptive in terms of concepts/behaviors/constructs g. Overemphasis on scientism	-Theoretical pluralism (R, P) -Interdisciplinary collaboration (R, P) -Skeptics & critics (R, P) -Diverse methodologies (R, P) -Methodological rigor (R) -Evaluative rigor (P) -Methodological experts (R) -Balanced reporting & responsible claims (R) -Balanced communication & responsible claims (P)
2.3	Poor research designs	There is an overreliance on cross-sectional and correlational studies, leading to exaggerated causal claims.	a. Over-reliance on cross-sectional designs b. Inference of causality c. Paying lip service to limitations d. Everything always correlating	-Methodological expertise and professional development (R) -Scientific literacy & professional development (P) -Diverse methodologies (R, P) -Methodological rigor (R) -Evaluative rigor (P) -Methodological experts (R) -Context-sensitive design (R, P) -Balanced reporting & responsible claims (R) -Balanced communication & responsible claims (P)
2.4	Lack of robust research approaches	PP neglects a broader range of research methods, including qualitative, mixed-method, longitudinal, and experimental designs.	a. Lack of qualitative/mixed-method/experimental research	-Diverse methodologies (R, P) -Methodological rigor (R) -Evaluative rigor (P) -Methodological experts (R)
**Theme 3: Positive psychology is a pseudoscience: poor replication and lacking evidence**.				
	*Positive psychology is a pseudoscience that makes false claims about benefits, over-exaggerates the implications of findings, is rife with confirmation bias, and important findings cannot be replicated*.			
3.1	Poor replication	PP has issues of replicability, a perceived lack of empirical support for interventions, and potential contradictions with established findings in other fields.	a. Lack of replication/verification b. Evidence from other fields (e.g., medicine) contradicted by claims	-Methodological expertise and professional development (R) -Scientific literacy & professional development (P) -Intellectual humility (R, P) -Theoretical pluralism (R, P) -Methodological rigor (R) -Evaluative rigor (P) -Methodological experts (R) -Replicability & transparency (R) -Theory-driven evaluation (P) -Iterative testing (P) -Context-sensitive design (R, P) -Balanced reporting & responsible claims (R) -Balanced communication & responsible claims (P)
3.2	Pseudoscience: lack of evidence	PP researchers make false claims that overstate benefits with a lack of empirical support for some theories. There is tautological reasoning and trivial conclusions that state the obvious, confirmation bias and projection of pre-existing beliefs, the use of complex statistical techniques that may obscure results, and circular reasoning with unjustified generalizations.	a. False claims about benefits b. No empirical evidence to support theories/claims c. Intellectual dishonesty d. Socially constructed illusions e. Neural correlates not promising results f. Shelter sought in statistics and neuroscience g. Confirmation bias h. Tautological statements, superficial knowledge, and obvious conclusions i. Common sense and traditional wisdom j. Not self-correcting k. Circular reasoning	-Intellectual humility (R, P) -Skeptics & critics (R, P) -Interdisciplinary collaboration (R, P) -Theoretical pluralism (R, P) -Context-sensitive design (R, P) -Evidence-based interventions (P) -Iterative testing (P) -Balanced reporting & responsible claims (R) -Balanced communication & responsible claims (P)
**Theme 4: Positive psychology lacks novelty and self-isolates from mainstream psychology**.				
	*Brings nothing new to the proverbial table and it willfully created a divide between “negative” psychology and the study of “optimal human functioning.”*			
4.1	Lack of novelty	Reliance on repetitive concepts from past theorists and shared methodologies common across psychological disciplines. The tendency to make exaggerated claims about the impact of findings without solid empirical support. The need for more rigorous scrutiny and open debate.	a. Questionable usefulness b. No new models or paradigms c. No innovative techniques or new methods d. Overlapping with other paradigms (e.g., cognitive behavioral therapy (CBT) and humanistic psychology) e. Current trends (fashion) vs. meaningful progress f. Over exaggeration of findings and false claims made g. No critical reflection h. Dispensable and discourse exhausted	-Intellectual humility (R, P) -Historical roots (R, P) -Theoretical pluralism (R, P) -Construct & conceptualization rigor (R, P) -Skeptics & critics (R, P) -Interdisciplinary collaboration (R, P) -Cultural perspectives (R, P) -Balanced reporting & responsible claims (R) -Balanced communication & responsible claims (P)
4.2	Lack of integration with mainstream psychology	PP is a siloed discipline that ignores its historical roots, fails to integrate the usefulness of the “negative,” and fails to build on what's being done in the rest of psychology and in other fields.	a. Isolation from other fields b. No incorporation of what has been learnt c. Mainstream psychology viewed as negative d. Shallow interpretations of reality and unreflective on critiques e. Usefulness of the negative ignored f. Barriers to discovery g. Historical origins of discipline ignored h. Humanistic vs. positive psychology divide	-Intellectual humility (R, P) -Historical roots (R, P) -Theoretical pluralism (R, P) -Construct & conceptualization rigor (R, P) -Complexity in the positive (R, P) -Skeptics & critics (R, P) -Interdisciplinary collaboration (R, P)
**Theme 5: Positive psychology is a decontextualized neo-liberalist ideology that causes harm**.				
	*Classified as a new-liberalist ideology where optimal functioning and flourishing are seen as an individual enterprise and a consequence of one's own life choices. It neglects the role of context/environment in understanding positive phenomena. This causes harm*.			
5.1	Decontextualization	PP is a decontextualized intellectual endeavor that neglects the importance of cultural, societal, and environmental factors, is biased towards Western values as universal, and ignores the role of context, leading to accusations of cultural insensitivity.	a. Role of context neglected b. WEIRD enterprise (cultural bias: Western values as “universal”) c. Not value neutral d. Social factors neglected e. Gender, class, ethnicity, power relations, society, institutions, and situations (bias) f. Social transformation g. Ignorant and insensitive h. Universalism and objectivism i. Norms and values ignored j. Justification of lack of findings with context	-Self-awareness & critical self-evaluation (R, P) -Cultural competence & power dynamics (R, P) -Intellectual humility (R, P) -Methodological expertise and professional development (R) -Theoretical pluralism (R, P) -Interdisciplinary collaboration (R, P) -Skeptics & critics (R, P) -Participant involvement (R, P) -Cultural perspectives (R, P) -Research-practice collaboration (R, P) -Iterative testing (P) -Context-sensitive design (R, P) -Ethical responsibility (R, P) -Accessible dissemination (R, P)
5.2	Neo-liberalist ideology	PP has a neo-liberalist ideology that attributes individual success and problems to personal choices, facilitated by capitalism, while ignoring social and environmental factors. This shifts responsibility for happiness onto individuals, promoting control and dependency rather than addressing systemic issues and providing liberating knowledge.	a. Self-fulfillmen b. Conceptual and political c. Ideology d. Control e. External goals f. Exoneration from social responsibilities	-Self-awareness & critical self-evaluation (R, P) -Cultural competence & power dynamics (R, P) -Intellectual humility (R, P) -Methodological expertise and professional development (R) -Theoretical pluralism (R, P) -Interdisciplinary collaboration (R, P) -Skeptics & critics (R, P) -Participant involvement (R, P) -Cultural perspectives (R, P) -Context-sensitive design (R, P) -Ethical responsibility (R, P) -Balanced reporting & responsible claims (R) -Balanced communication & responsible claims (P)
5.3	Causing harm	PP can cause harm by pathologizing normal behaviors, setting unrealistic expectations, and fostering an irrational obsession with happiness. PPIs can lead to negative consequences that need care. PP can be culturally biased and context-insensitive, reinforcing gender and cultural stereotypes, ignoring systemic oppression, and perpetuating power imbalances.	a. “Normal” human behavior pathologized b. Unrealistic goals and expectations c. Irrational happiness/obsession with happiness d. Overemphasis of optimism e. Therapy/medication increased f. Stigma created g. Reinforcement of gender/cultural stereotypes h. Social change limited i. No consideration of women j. Status quo maintained k. Alienation and oppression l. Marginalization of groups m. Racism ignored n. Transgender ignored	-Self-awareness & critical self-evaluation (R, P) -Cultural competence & power dynamics (R, P) -Complexity in the positive (R, P) -Interdisciplinary collaboration (R, P) -Skeptics & critics (R, P) -Participant involvement (R, P) -Cultural perspectives (R, P) -Research-practice collaboration (R, P) -Iterative testing (P) -Context-sensitive design (R, P) -Ethical responsibility (R, P) -Accessible dissemination (R, P) -Balanced reporting & responsible claims (R) -Balanced communication & responsible claims (P)
**Theme 6: Positive psychology is a capitalistic venture**.				
	*It's a capitalistic tool that aims to commercialize “positivity” as a means to further facilitate individualism, consumerism, and the medicalization of positive experiences*.			
6.1	Commercialization	PP is a capitalistic venture that promotes individualism and consumerism by commercializing happiness and positive experiences, creating a market that drives profits on the impossible dream of obtaining happiness.	a. Capitalistic venture b. Needing positive psychology to be happy c. Market of happiness/medicalization d. Market of impossible dreams e. Markets for goods and services	-Research-practice collaboration (R, P) -Accessible dissemination (R, P) -Balanced reporting & responsible claims (R) -Balanced communication & responsible claims (P) -Conscious funding choices (R, P)

The critiques and criticisms categories, sections, elements, and overarching definitions were first published Van Zyl et al. (2023). The table has been reproduced and adapted with permission. The section-level descriptions and the checklist representation columns are new to this document and includes the content from both the researcher and practitioner checklists. R stands for the researcher version; P stands for the practitioner version. PP stands for positive psychology.

## Data Availability

The original contributions presented in the study are included in the article, further inquiries can be directed to the corresponding author.

## Appendix B

**How to use the checklists**

The two checklists are intended for PP professionals, including anyone researching, designing, or evaluating PPIs. By using the checklists at various stages and for different purposes, PP professionals can enhance the rigor, effectiveness, and impact of their work:

- Researchers:
  ° Creating Research Proposals: When beginning the process of crafting a research proposal or planning a new study, researchers can use their version of the checklist to ensure that all critical elements that mitigate the critiques of the field are considered and properly addressed.
  ° Critiquing Proposals and Studies: Researchers can refer to the checklists when evaluating research proposals or completed studies including before submitting a manuscript for publication. This can help identify potential weaknesses or areas for improvement and ensure the study does not propagate the critiques of the field.
  ° Journal Review Process: Journals reviewing studies can use the checklists as a standard to assess the robustness and completeness of submitted research, while being open-minded to various ways of knowing, innovations, and resource constraints

- Practitioners:
  ° Planning New Interventions: When starting to plan a new intervention, practitioners can use their version of the checklist to guide the development process, ensuring they mitigate the critiques of the field.
  ° PPI Implementation: At any stage of PPI planning or implementation, practitioners can consult the checklists to make sure their approach is appropriate.

- Evaluators:
  ° Evaluating PPIs: Evaluators can refer to the checklists when conducting evaluations of PPIs. This helps ensure that their evaluations are thorough, systematic, and based on established standards. Evaluators can also use the checklists for evaluation capacity-building.

- Across groups:
  ° Ongoing Professional Development: All users can revisit the checklists regularly to ensure they are mitigating the critiques of the field. As the checklists are iterated, this review process of continued professional development will continue to be important.

The checklists are merely conceptual frameworks and are not prescriptive. Here is a list of points for using the checklists:

- Timing: Start wherever you are. Users can review the appropriate checklist in its entirety and then reference the area(s) most relevant for their timing and circumstances.
- Flexibility: The checklists can be used flexibly; their linear presentation does not imply they must be followed comprehensively or in order. Adapt the checklist based on the specific needs of your project. This is a guide, not a prescriptive set of rules.
- Dynamic Nature: Recognize that the structured nature of checklists may not fully accommodate the dynamic nature of real-world studies and interventions. Users are encouraged to adjust, skip, or rearrange checkpoints as necessary.
- Avoid Tunnel Vision: Stay open to new opportunities and adapt goals as needed to avoid becoming too focused on the specific structure of the checklist(s).
- Inclusivity: Incorporate diverse perspectives and stakeholders to enhance the robustness of your use of the checklists.
- Expertise: Use the checklist to complement, not override, your knowledge and experience. Not everything will be appropriate for every context.
- Practical Considerations: Implementing some recommendations, like methodological diversity and interdisciplinary collaboration, may be challenging in resource-constrained settings. In the absence of specialized expertise, take alternative approaches or adaptations where necessary.
- Barriers to Implementation: Consider potential barriers to implementation, such as gatekeeping and resource limitations.
- Different Ways of Knowing: Acknowledge that there are different ways of knowing that will impact how the checklists are used in your context.

## Appendix C

**Figure A1****. Checklist #1: Strategies for researchers to design future positive psychology studies that mitigate the critiques and criticisms of the field**. This checklist has been developed for researchers when designing positive psychology studies to mitigate the critiques and criticisms of the field. It can be used throughout the study process, from pre-study through the dissemination of findings. For more detail on each checkpoint, users can review the body of this paper. This is version 1.

**Researcher's self-reflection & development**

□ **Self-awareness & critical self-evaluation:** Cultivate a consistent practice of reflexivity and self-evaluation to understand how research paradigm, positionality, personal biases, values, and foundational beliefs influence your research questions, methodologies, and interpretations. Actively challenge the assumptions underpinning your research.
□ **Cultural competence & power dynamics:** Enhance your cultural competence to conduct research that is respectful of and sensitive to diverse cultural contexts. Recognize the values and foundational beliefs of other cultures that may impact your areas of study. Recognize and conscientiously navigate the power structures and societal dynamics that may impact the direction and reception of your work.
□ **Intellectual humility:** Approach your research with humility, recognizing the limits of your knowledge and expertise. Remain open to new evidence that may support or challenge your beliefs, your theories, or your findings.
□ **Methodological expertise & professional development:** Stay informed about the latest research findings and maintain a critical stance towards new trends that may not have a strong evidence base. Invest in your methodological expertise. Employ a healthy skepticism that promotes regular inquiry and re-evaluation of your research. Invest in your methodological expertise to employ the appropriate method well. Actively participate in scholarly communities, like the International Positive Psychology Association (IPPA), to foster knowledge exchange and intellectual growth.

**Conceptual foundations**

□ **Historical roots:** Deepen your understanding of the origins and evolution of positive psychology and its positionality within the wider realm of psychological science. Demonstrate how your work has built upon the foundational scholarship that pre-dated and/or is outside of positive psychology (e.g., humanistic psychology, ancient philosophy).
□ **Theoretical pluralism:** Integrate theoretical perspectives from outside of positive psychology and incorporate multi-level systems-thinking approaches (e.g., individual, group, organizational) to address the complexity of your research and wellbeing more broadly. Then identify and articulate the theoretical frameworks and empirical evidence that informs your research.
□ **Construct & conceptualization rigor:** Perform comprehensive literature reviews and seek alternative perspectives when choosing constructs and measures for your studies. Avoid using different terms for similar constructs (jangle fallacy) or conflating different constructs under the same label (jingle fallacy).
□ **Complexity in the positive:** Clearly articulate what constitutes the “positive” in positive psychology and how your work contributes to it. Integrate with other fields/disciplines and explore beyond simple positive/negative dichotomies to further incorporate complexity.

**Multidisciplinary perspectives**

□ **Interdisciplinary collaboration:** Foster dialogue and collaboration with experts from diverse fields/disciplines (e.g., medicine, sociology, anthropology, political science, and social sciences) to enrich your understanding and perspective on wellbeing and of your research topic more specifically.
□ **Methodological experts:** Collaborate with methodological experts and utilize peer review processes to refine and validate your research design and analysis.
□ **Skeptics & critics:** Anticipate and seek out the perspectives of skeptics who might dismiss your research questions and ultimate findings as trivial or self-evident. Integrate these critical perspectives to strengthen your research design and include these counter arguments when reporting findings.
□ **Participant involvement:** Consider ways to collaborate, consult, or solicit feedback from the intended population or participants.
□ **Cultural perspectives:** Incorporate cross-cultural or culturally relevant perspectives in your research team and participant pool when appropriate (e.g., non-WEIRD and indigenous populations).
□ **Research-practice collaboration:** Consider working in tandem with practitioners to further close the research-practice gap. Collaborate with practitioners to help them translate their work with evidence-informed approaches. Gain insights and bright spots from practitioners that can inform research streams.

**Methodological rigor**

□ **Diverse methodologies:** Utilize a range of research methods, including both quantitative and qualitative approaches, to capture the multi-faceted nature of positive psychological phenomena. (e.g., longitudinal and experimental designs can demonstrate causality and changes over time; mixed methods and qualitative designs can capture richness and nuance; ecological momentary assessment can avoid self-report bias, participatory action-research can co-construct alongside marginalized communities). Ultimately, justify the chosen methodological approach.
□ **Methodological rigor:** Maintain methodological precision for research design, including adhering to method-specific standards (e.g., Cochrane's assessment of bias for randomized controlled trials).
□ **Psychometric rigor:** When introducing new constructs, conceptualizations, or measures/operationalizations, collaborate and seek consensus with other researchers and perform thorough validation and reliability testing.
□ **Replicability & transparency:** Allow for scrutiny, constructive criticism, and replication by other researchers. Pre-registering studies (e.g., the Open Science Framework). Publish null or inconclusive findings, full datasets, methodologies, and statistical analyses. Conduct replication studies of your own.

**Customization to context and ethical responsiveness**

□ **Context-sensitive design:** Ensure that your research design is tailored to the specific sample and context. Consider individual and cultural differences, as well as social, cultural, and systemic factors.
□ **Ethical responsibility:** Be proactive in addressing ethical concerns. Assess the broader, long-term impact of your research and findings, considering the potential for harm and unintended consequences. Ensure that your research promotes diversity, equity, and inclusivity.
□ **Conscious funding choices:** Carefully consider the sources of funding to avoid commercial interests that might skew the research toward capitalistic ventures.

**Balanced and accessible reporting**

□ **Balanced reporting & responsible claims:** Base conclusions on solid research findings. Avoid overgeneralization or overstating significance. Report limitations. Use causal language judiciously. Refrain from projecting individual results as representative of broader systemic outcomes. Allow for scrutiny and constructive criticism.
□ **Accessible dissemination:** Strive to make your findings widely accessible by minimizing cost and using clear language. Educate practitioners, the media, and the public, partner with them to ensure accurate representation of your research.

## Appendix D

**Figure A2****. Checklist #2: Strategies for practitioners and evaluators to develop and evaluate future positive psychology interventions that mitigate criticisms**. This checklist has been developed for practitioners when developing positive psychology interventions to mitigate the critiques and criticisms of the field. It can be used throughout the process, from pre-development and design through implementation and evaluation. Evaluators can also use this checklist throughout the process of evaluating positive psychology interventions. For more detail on each checkpoint, users can review the body of this paper. This is version 1.

**Practitioner self-reflection & development**

□ **Self-awareness & critical self-evaluation:** Cultivate a consistent practice of reflexivity and self-evaluation to understand how your positionality, personal biases, values, and foundational beliefs influence your intervention design and dissemination. Actively challenge the assumptions underpinning your approach.
□ **Cultural competence & power dynamics:** Develop cultural competence to ensure interventions are respectful and appropriate across diverse contexts. Recognize the values and foundational beliefs of other cultures that may impact how others would receive your intervention. Recognize and conscientiously navigate the power structures and societal dynamics that may impact the direction and reception of your work.
□ I**ntellectual humility:** Approach your intervention with humility, recognizing the limits of your knowledge and expertise. Remain open to new insights that may support or challenge your beliefs and practice.
□ **Scientific literacy & professional development:** Stay informed about the latest research findings and maintain a critical stance towards new trends that may not have a strong evidence base. Employ a healthy skepticism that promotes regular inquiry and re-evaluation of your practice. Actively participate in scholarly communities, like the International Positive Psychology Association (IPPA), to foster knowledge exchange and intellectual growth.

**Conceptual foundations**

□ **Evidence-based interventions:** Base interventions on strong theoretical frameworks, empirical evidence, and/or insights from practice, outlining clear mechanisms of change that explain how and why they work (e.g., a theory of change or logic model).
  ° **Theoretical pluralism:** Integrate theoretical perspectives from outside of positive psychology, incorporate multi-level approaches (e.g., individual, group, organizational), and explore existing interventions across disciplines to design a robust intervention and address the complexity of wellbeing.
  ° **Historical roots:** Deepen your understanding of the origins and evolution of positive psychology and its positionality within the wider realm of psychological science.
□ **Construct choice & terminology:** Perform comprehensive literature reviews and seek alternative perspectives when choosing constructs and concepts for your intervention. Try to be consistent to common constructs/terms when possible. Avoid using different terms for similar constructs (jangle fallacy) or conflating different constructs under the same label (jingle fallacy). When adapting terminology to fit your context, still note the common research terms to build capacity and reduce the science-practice gap.
□ **Complexity in the positive:** Clearly articulate what constitutes the “positive” in positive psychology and how your work contributes to it. Integrate with other fields/disciplines and explore beyond simple positive/negative dichotomies to further incorporate complexity.

**Multidisciplinary perspectives**

□ **Interdisciplinary collaboration:** Collaborate with experts from various fields (e.g., medicine, sociology, anthropology, political science, and social sciences) to enrich your understanding and perspective on wellbeing and to enhance your intervention design and relevance.
□ **Skeptics & critics:** Anticipate and seek out the perspectives of skeptics who might dismiss your intervention as trivial or self-evident. Integrate these critical perspectives to strengthen your intervention design and include these counter arguments in your intervention.
□ **Participant involvement:** Engage with participants and communities for feedback and shared decision-making.
□ **Cross-cultural perspectives:** Incorporate cross-cultural perspectives in your design team and participant pool when appropriate (e.g., non-WEIRD and indigenous populations).
□ **Research-practice collaboration:** Consider working in tandem with researchers to further close the research-practice gap. Work with researchers to design interventions that can be empirically tested; this collaborative approach can help you ground your intervention in solid research while contributing to the scientific literature. Likewise, offer researchers new insights from your real-time work in practice.

**Methodological rigor in evaluation**

□ **Theory-driven evaluation**: Align assessment methods with the intervention's theoretical framework for coherent evaluation.
□ **Evaluative rigor:** Utilize existing measures and evaluation practices in your evaluation. Work with expert researchers and evaluators that will adhere to methodological rigor and standards.
□ **Diverse methodologies:** Utilize a range of research methods when evaluating, including both quantitative and qualitative approaches, to capture the multi-faceted nature of positive psychological phenomena. (e.g., longitudinal and experimental designs can demonstrate causality and changes over time; mixed methods and qualitative designs can capture richness and nuance; ecological momentary assessment can avoid self-report bias, participatory-action research can co-construct alongside marginalized communities). Ultimately, justify the chosen methodological approach.
□ **Iterative testing:** Refine interventions through piloting, monitoring, regular assessments, feedback loops, and continuous improvement.

**Customization to context and ethical responsiveness**

□ **Context-sensitive design:** Ensure that your intervention design is tailored to the specific audience and context. Consider individual and cultural differences, as well as social, cultural, and systemic factors. Acknowledge and work to understand structural barriers that participants may face in their pursuit of wellbeing.
□ **Ethical responsibility:** Be proactive in addressing ethical concerns. Assess the broader impact of your intervention's impact, considering the potential for harm and unintended consequences. Ensure that your intervention promotes diversity, equity, and inclusivity. Review ethical guidelines for practitioners.
□ **Conscious funding choices:** Carefully consider the sources of funding to avoid commercial interests that might skew the research towards capitalistic ventures.

**Balanced & accessible communication**

□ **Balanced communication & responsible claims:** Communicate both the benefits and the limitations of the intervention's intended outcomes without overstating, overpromising, or overgeneralizing. Use causal language judiciously. Refrain from projecting individual results as representative of broader systemic outcomes. Allow for scrutiny and constructive criticism.
□ **Accessible dissemination:** Strive to make your intervention accessible by minimizing cost, using clear language, and addressing structural barriers to wellbeing. Partner with the media to ensure accurate representation of the scientific evidence that backs your intervention.
